# Bioinspired Device Improves The Cardiogenic Potential of Cardiac Progenitor Cellst

**DOI:** 10.22074/cellj.2021.7232

**Published:** 2021-03-01

**Authors:** Zahra Shams, Babak Akbari, Sarah Rajabi, Nasser Aghdami

**Affiliations:** 1 Department of Life Science Engineering, Faculty of New Sciences and Technologies, University of Tehran, Tehran, Iran; 2 Department of Cell Engineering, Cell Science Research Centre, Royan Institute for Stem Cell Biology and Technology, ACECR, Tehran, Iran; 3 Department of Regenerative Medicine, Cell Science Research Centre, Royan Institute for Stem Cell Biology and Technology, ACECR, Tehran, Iran

**Keywords:** Aligned Scaffold, Cardiac Progenitor Cells, Cardiac Tissue Engineering, Mechanical Simulation

## Abstract

**Objective:**

Functional cardiac tissue engineering holds promise as a candidate approach for myocardial infarction. Tissue engineering has emerged to generate functional tissue constructs and provide an alternative means to repair and regenerate damaged heart tissues.

**Materials and Methods:**

In this experimental study, we fabricated a composite polycaprolactone (PCL)/gelatine electrospun scaffold with aligned nanofibres. The electrospinning parameters and optimum proportion of the PCL/ gelatine were tested to design a scaffold with aligned and homogenized nanofibres. Using scanning electron microscopy (SEM) and mechanophysical testes, the PCL/gelatine composite scaffold with a ratio of 70:30 was selected. In order to simulate cardiac contraction, a developed mechanical loading device (MLD) was used to apply a mechanical stress with specific frequency and tensile rate to cardiac progenitor cells (CPCs) in the direction of the aligned nanofibres. Cell metabolic determination of CPCs was performed using real-time polymerase chain reaction(RT-PCR).

**Results:**

Physicochemical and mechanical characterization showed that the PCL/gelatine composite scaffold with a ratio of 70:30 was the best sample. In vitro analysis showed that the scaffold supported active metabolism and proliferation of CPCs, and the generation of uniform cellular constructs after five days. Real-time PCR analysis revealed elevated expressions of the specific genes for synchronizing beating cells (MYH-6, TTN and CX-43) on the dynamic scaffolds compared to the control sample with a static culture system.

**Conclusion:**

Our study provides a robust platform for generation of synchronized beating cells on a nanofibre patch that can be used in cardiac tissue engineering applications in the near future.

## Introduction

Cardiovascular diseases are one of the leading causes of death worldwide, with almost 40% of morbidity and mortality in both developed and developing countries (1). In 2013, more than 17.3 million deaths were attributed to cardiovascular diseases and this number is expected to exceed 23.6 million by 2030 (2). Various types of treatments used in patients diagnosed with heart failure include non-invasive methods (medications) and invasive methods such as angioplasty, ventricular assist devices, pacemakers, and eventually heart transplantation (3, 4). In these methods, the main goal is to help the heart to partially restore cardiac function and prevent disease progression, despite the loss of some cardiac cells. None of these procedures repair lost tissue. The heart transplant, which is considered an end-stage treatment, has many limitations due to the lack of donors and complications associated with immune suppressive treatments (5).

Therefore, scientists have focused on modern approaches such as cell therapy and tissue engineering (4). In cell therapy, viable cells can be directly injected into the infarcted area or arterially infused (6). These procedures were not very successful because only 15% of the cells could reach the intended site following the arterial injection. In the case of direct injections also, only a small number of injected cells could function properly due to the lack of an appropriate scaffold for feeding and growth (7, 8). 

In this regard, scaffolds that contain cardiac progenitor cells (CPCs) can function with high productivity in therapeutic procedures (9-11). The suitable scaffold for cardiac tissue engineering should mimic the natural extracellular matrix (ECM) of cardiomyocytes (12, 13). In addition to its appropriate adhesion and strength, as heart tissue is imposed under tension loadings of continuous and cyclic contraction and expansion, a suitably fabricated scaffold should withstand this level of tension to provide mechanical support for cardiac cells during the repair period (14-16). Heart muscle has a Young's elastic modulus range from 10-20 kPa in diastole with a tension rate of <10%. At the end of diastole, Young's modulus will increase to 50 in normal cardiac muscle and 200– 300 kPa in the damaged heart. Therefore, an elastomer scaffold such as polycaprolactone (PCL) has a very appropriate application in contractive cycles of cardiac tissue (17). PCL has good mechanical properties and a controllable degradability rate (17, 18). However, intrinsically it is hydrophobic and cannot provide the appropriate conditions for cell adhesion. Therefore, it is better to integrate a natural scaffold such as gelatine with PCL to produce a composite with better adhesion and mechanical strength. The composite ratio of these polymers is very important (17, 18).

An important issue with the transplantation of cellseeded scaffolds to the infarcted area is that the seeded cells lack the ability to regulate themselves with other natural cardiomyocytes during beating. Therefore, they will cause heart arrhythmia (19). Exposing CPCs to mechanical loadings at a frequency and tension similar to natural tissues will increase expressions of the genes related to cell contraction and synchronization (20-22). 

Mechanical loading transmission in a specific, direct way has a greater impact on the speed and quality of the conduction (23, 24). Thus, in this study, we designed a two-dimensional (2D) aligned nanofibre composite scaffold that was fabricated with the appropriate rate of two PCL and gelatine polymers using electrospinning techniques with a rotating mandrel. We exposed the scaffold to mechanical loading in the direction of the parallel nanofibres at specific frequency and tension rates created by a mechanical loading device (MLD). Therefore, we simulated the conditions of natural cardiac cells as much as possible *in vitro*. Although numerous researches have been conducted that imposed mechanical and electrical loadings to the scaffolds with cells (25-27), mechanical loadings have not been directly imposed on 2D anisotropic electrospun scaffolds in the direction of parallel nanofibres that contain CPCs. 

In the present study, mechanical loading was transferred in the direction of the aligned nanofibres; therefore, the interactive effects of anisotropy and scaffold tension induced the human cardiac progenitor cells (hCPCs) to differentiate into cardiac cells.

## Materials and Methods

### Scaffold fabrication

In this experimental study, a mixture of formic acid and acetic acid (7:3) (Sigma Aldrich Corporation) was used as solvent to obtain a 14% (wt %) polymer solution (28). The proportion of formic acid was Greater in the solvent because of its high dielectric constant (29). To achieve optimum electrospinning parameters for an aligned and homogenized nanofibre scaffold, PCL (mw: 80 000 g/mol, Sigma Aldrich) and porcine skin gelatine type A (Sigma Aldrich) polymers were mixed at a PCL/gelatine ratio of 70:30 and added to the solvent. The solvent was shaken on a stirrer at 500 rpm for one hour without heat. Electrospinning techniques were applied to fabricate the scaffold from the prepared solution. The Mandrel rotation technique was used to have aligned nanofibres. To obtain optimum electrospinning parameters, we used varied feeding ratios, needle distance to collector, voltage, and the Mandrel rotation speed [(30), Table 1]. The samples were prepared for scanning electron microscopy (SEM) imaging to assess the morphological features, level of homogeneity, and direction of the nanofibres. To obtain the image from a polymer surface using electron radiation, a gold coating should be applied in order to make a conductive surface. The samples were imaged at 2000 V.

After specifying the appropriate electrospinning parameters, we assessed the different ratios in terms of hydrophilicity and mechanical strength. To achieve this purpose, three scaffold samples were fabricated with PCL/gelatine composite ratios of 80:20, 70:30 and 60:40 according to optimum electrospinning parameters.

**Table 1:** Different electrospinning parameters of the polycaprolactone (PCL)/gelatine (70:30) at a 14% (wt%) concentration at room temperature


Sample	Rate (ml/hour)	Distance: Needle to collector (cm)	Voltage (kV)	Collector speed (RPM)	Electrospinning Time (minutes)

A1	0.1	15	17	2000	10
A2	0.2	12	15	2000	15
A3	0.1	12	12	1800	10
B1	0.3	10	17	1500	20
B2	0.3	10	17	2000	20
B3	0.3	10	17	2500	20


According to SEM studies, the scaffold diameter distribution and discrepancy levels were compared using SPSS software in order to detect those composites with the highest homogeneity. Hydrophilicity was studied using contact angle tests in the three scaffold composites with different ratios. The static contact angle was measured with the sessile drop technique by placing a 3 µl droplet on a polymer surface to obtain images with a camera when the droplet stabilized. Mechanical strength was compared among the three composite ratios using an Instron TMSM (Instron^®^, UK). First, the length and diameter values were measured in the samples, then a tension force was imposed on samples in the direction of the nanofibres with a strain rate of 5 mm/minute (31). After five repetitions for each sample, tension-strain curves were plotted and compared, and the best electrospinning parameters and polymer ratio to fabricate the main scaffold were chosen.

### Cell viability assessment 

Human cardiac progenitor cells (hCPCs) were purchased from Royan Institute (code no. RSCB0180, Tehran, Iran). The cells were cultured in a culture medium that included Iscove’s modified Dulbecco’s medium (IMDM, Sigma), 1% L-glutamine (Invitrogen), non-essential amino acids (Invitrogen), penicillin/streptomycin (Invitrogen) and 10% fetal bovine serum (FBS, Gibco). The medium was changed every two days. Cells were passaged with 0.025% trypsin/EDTA (Gibco) for 3 minutes at 37˚C. Gelatine is a hydrophilic polymer, and the nanofibre morphology could be destroyed in aqueous fluids. Therefore, the nanofibre was cross-linked by treatment with 25% glutaraldehyde (Merck, Germany) in a desiccator for six days. Approximately 3000 cells were cultured on scaffolds for two, four, and six days. The MTS (Promega, G5421) assay was performed to determine cytotoxicity at the specified time and according to the manufacturer’s instructions. Absorbance was measured at 490 nm using an enzyme-linked immunosorbent assay (ELISA) plate reader (Thermo Scientific Multiscan Spectrum). The PCL scaffold was used as the control group.

### Mechanical loading device fabrication

**Fig.1:**
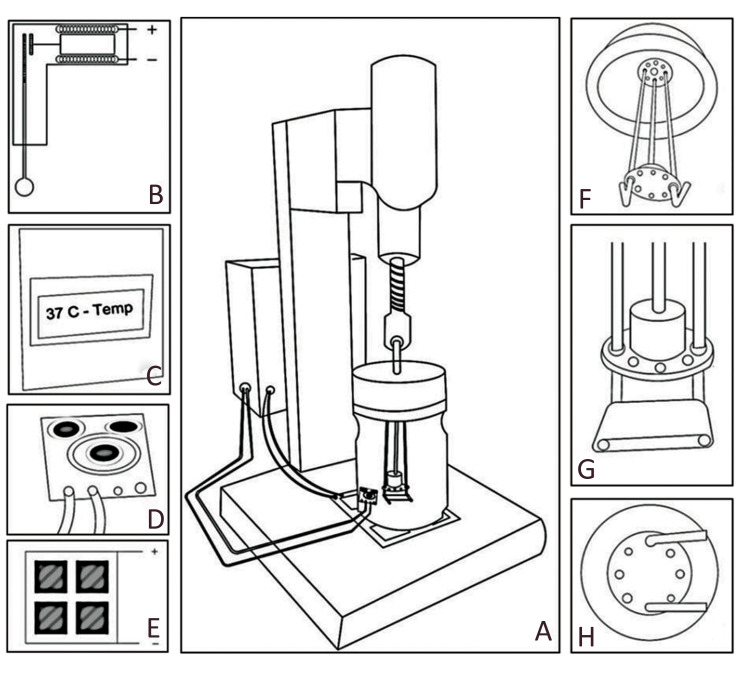
Assembly simulation of the mechanical loading device (MLD). **A. **MLD schematics. **B. **Armature for applying the mechanical load. **C. **Frequency board and heat controller in a box. The LCD embedded in the box displays the off/on mode of the heater and the temperature of the culture media in the bottle. **D. **Non-contact infrared temperature sensor measures the media temperature. **E. **Steel holder, which includes a 10×10 cm heater at the bottom of the steel plate. **F.** Steel bases attached to the door. The steel shaft attached to the armature core causes two bases to open and close at a specific frequency. **G.** Electrospun scaffold located on the steel bases. **H.** Teflon piece with eight holes (four optional axes of force). R=2 cm.

To mimic the morphological and functional properties of native cardiomyocytes in the body, the cells that were seeded on the scaffold should be exposed to mechanical loading according to a normal heart beat (32-34). Therefore, a device was designed where its applied force could be controlled with specific tension rate and frequency (Fig .1). This device was designed with a stainless steel body and an armature that could be run according to the frequency and force values determined, and confirmed by a frequency generator board. The armature included a coil and core placed inside. The core was connected to a metal shaft. The shaft passed through a hole in the bottle lid and Connected to two steel bases inside the bottle. The scaffold was placed on these bases. The coil could generate a strong magnetic field with an imposed voltage of 5 V (35), where the core would move in the direction of the magnetic field and create a distance between two bases. After the imposed voltage is disconnected, the bases return to the initial locations. The distance between two bases and the time switch were considered to be capable of generating a tension force with a 10% strain and frequency of 1 Hz (25, 33, 35, 36) in the scaffold, which was adhered to two bases with antibacterial silicone glue. All parts that were in direct and indirect contact with the scaffold were made of Teflon and steel so they could be autoclaved.

**Fig.2:**
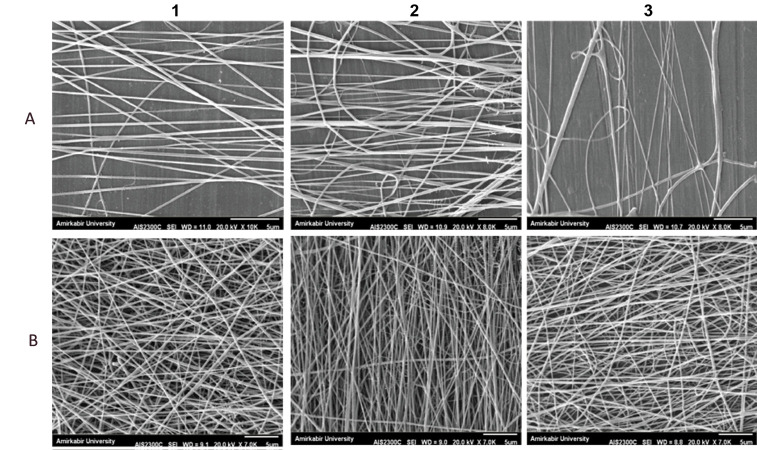
Scaffold homogeneity analysis by scanning electron microscopy (SEM) micrographs of the aligned polycaprolactone (PCL)/gelatine (70:30) with different electrospinning parameters (Table 1) for the A1, A2, A3, B1, B2 and B3 samples.

The voltage input of the armature was turned on and off by a frequency generator board (Fig .1, See supplementary online information at www.celljournal.org). The scaffold could be exposed to a 10% strain per second. In order to set a temperature of 37˚C for cells without an incubator, we designed a system to control the temperature, which included a heating element, thermostat board, relay to turn the currents on and off, a non-contact thermal sensor for temperature control and a display device to represent temperature values during each moment (Fig .2, See supplementary online information at www.celljournal. org). The non-contact sensor mounted on the bottle sends infrared light into the cell culture medium, measures the returned light and determines the internal temperature of the culture container. If the recorded temperature is less or more than 37˚C, the sensor would send an on/off command to the thermostat board and relay. By using the mentioned system and continuous monitoring temperature on the display, we were assured that the temperature was appropriate for the cultured cells.

### Mechanical loading device experiment

CPCs were seeded on the nanofibre composite scaffold at 2×10^6^ per 2×2 cm^2^ and placed in the cell culture medium. One of the scaffolds was placed on the stainless-steel bases in the mechanical loading device (MLD). After three days in a fixed culture, the MLD was turned on and the cell-seeded scaffold was exposed to a mechanical loading in the direction of aligned parallel nanofibres at 10% elongation and frequency of 1 Hz for five days (33, 35). The temperature, humidity, oxygen, and pH were controlled in the culture environment to provide an appropriate environmental condition for cell growth and differentiation. To achieve this aim, the culture medium was changed daily to keep pH and oxygen levels at constant values (25). Also, the inner container temperature was monitored on an LCD display. During this period, the control scaffold was placed in an incubator with static culture medium. After applying the mechanical loading for five days, we prepared both scaffolds for imaging via SEM and gene expression analysis by real-time polymerase chain reaction (RT-PCR).

### Scanning electron microscopy images in the main and control scaffolds

Samples were fixed in 2% glutaraldehyde in 0.1 M PBS and left for 24 hours at 4˚C. The samples were washed with 0.1 M PBS and fixed in 1% OsO4 in 0.1 M PBS (pH=7.3) for 2 hours at 25˚C. The samples were subsequently dehydrated in a graded ethanol-water series to 100% ethanol, then allowed to completely dry. Finally, the samples were mounted on aluminium stubs and coated with a thin layer of gold. Cell morphology on the scaffolds was analysed with a scanning electron microscope (VEGA\TESCAN, Czech Republic) at an operating voltage of 15 kV.

### Determination of gene expression

In the present study, the expression levels of three genes (*TTN, MYH-6* and *GJA1*) were analysed and compared by real-time PCR (RT-PCR) in the dynamic and static culture conditions. RNA was extracted manually with TRIzol reagent (Ambion) and chloroform according to the manufacturer’s instructions. First strand cDNA synthesis was performed with a TaKaRa kit. Real-time PCR was performed using three cell samples: CSCs without any scaffolds, and cells seeded on scaffolds under static and dynamic conditions. Each condition was repeated four times (primer sequences in Table 1, Supplementary Information).

### Statistical analysis

All data were expressed as mean ± standard error mean. Statistical analysis was performed using one-way analysis of variance (ANOVA) followed by the appropriate post hoc test in Excel software (Microsoft Excel 2010). P values were considered significant at: *P <0.05, **P <0.01, and ***P<0.001.

## Results

### Scaffold characterization 

Figure 2 shows the SEM results of the composite scaffolds of the PCL/gelatine with a PCL to gelatine ratio of 70:30 that were created according to the mentioned electrospinning parameters in Table 1. According to the SEM images of all the samples, sample B2 was selected as the optimum sample.

**Fig.3:**
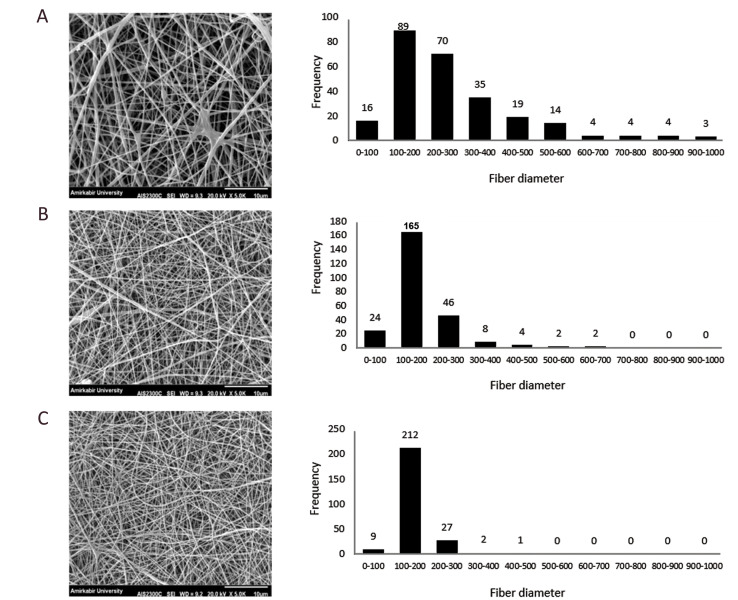
Scanning electron microscopy (SEM) micrographs and fibre diameter frequency of the random:** A.** polycaprolactone (PCL)/gelatine (60:40), **B. **PCL/ gelatine (70:30), and **C. **PCL/gelatine (80:20).

To obtain the optimum ratio of the composite scaffold, physicochemical and mechanical properties of different ratios of the PCL/gelatine (80:20, 70:30, 60:40) were evaluated. SPSS results and SEM images showed that as the gelatine ratio increased, the nanofibres showed higher heterogeneity [Fig.3, (18)].

**Fig.4:**
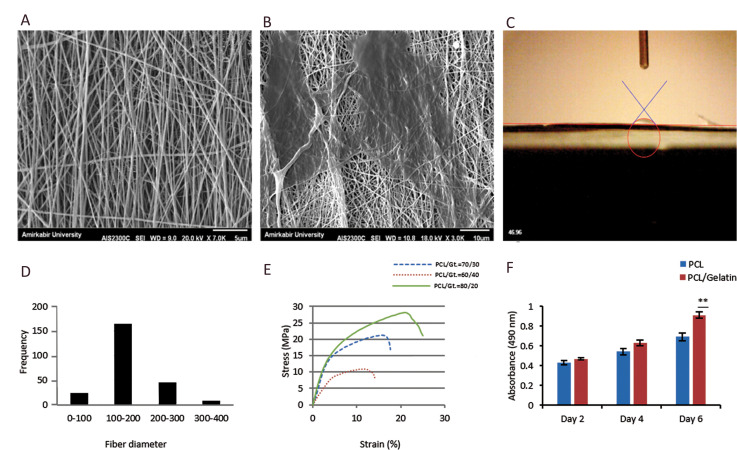
Mechanical, chemical and physical characteristics of the main scaffold. **A. **Scanning electron microscopy (SEM) image of the aligned polycaprolactone (PCL)/gelatine (70:30). **B.** SEM micrograph of cardiac progenitor cells (CPCs) on the scaffold at the fourth day of the static culture. **C.** Contact angle measurement. **D.** Fibre diameter frequency of the nanofibres (SPSS). **E. **Typical stress–strain curve of the PCL/gelatine 70:30 in comparison with the PCL/ gelatine 60:40 and 80:20 nanofibres. **F. **Cell proliferation and viability assays of the PCL/gelatine 70:30 nanofibres on days two, four, and six. *Significant differences and **P<0.01 versus control. PCL was used as the control (n=12).

The mechanical strength evaluation results demonstrated that the studied scaffolds tolerated a tension of 5 mm/ minute in the direction of the parallel nanofibres. The results were plotted in stress-strain figures for all samples (Fig .4E). Table 2 shows the results of the contact angle test and mechanical strength. Based on our results from SEM images and the contact angle and mechanical strength experiments, we selected the PCL/gelatine scaffold that had a composite ratio of 70:30 for further studies. The chosen scaffold had a contact angle of 46.96˚ and ultimate tensile strength of 22 MPa, which occurred at 17% elongation (Fig .4C). Since in this study, the scaffold was going to be exposed to 10% elongation, we concluded that our chosen scaffold could be used for the relevant tests.

**Table 2:** Young’s modulus and contact angle of the scaffolds with different ratios of Polycaprolactone and gelatine.


Omposite ratio (PCL/gelatine)	Young’s modulus (MPa)	Contact angle	Ultimate tensile strength (MPa)	Elongation at break point (%)

60:40	200	38.97˚	11	12
70:30	460	46.96˚	22	17
80:20	504	68.71˚	28	22


### Cell viability 

We performed the MTS assay to evaluate the cytotoxicity of the PCL/gelatine composite scaffold compared to the control PCL scaffold. As shown in Figure 4F, the number of cells on the scaffolds increased over time. At the fourth day, SEM images of the scaffolds indicated that the CPCs adhered to the aligned scaffold in the direction of the nanofibres (Fig .4B). 

### Cell proliferation in the static and dynamic samples

**Fig.5:**
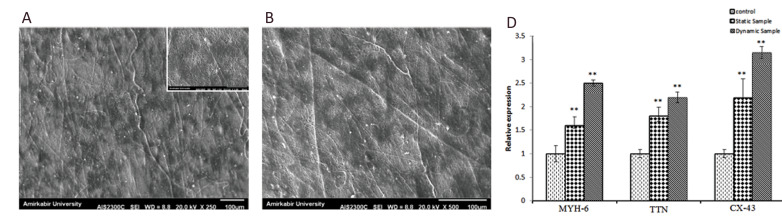
Cell morphology and gene expression on the scaffolds in the static and dynamic conditions. Scanning electron microscopy (SEM) micrographs of the cardiac progenitor cells (CPCs) on: **A. **dynamic sample after three days of static culture and five days of dynamic culture by the mechanical loading device (MLD) and **B. **static sample after eight days of static culture. **C. **Real-time PCR graph of the cardiac genes, *MYH-6, TTN* and *CX-43*, expressions in the control, static, and dynamic samples. *; Significant differences, and **P<0.01 versus control. Cardiac stem cells (CSCs) were used as the control (n=4).

We cultured CPCs on the selected composite scaffolds for three days. Then, we carried out mechanical loading on one of the scaffolds for five days using the MLD (Fig .1). Figures 5A, B show SEM images of these two samples.

### Gene expression results in the static and dynamic samples

Quantitative real-time PCR analysis was performed to evaluate expressions of the *TTN, MYH-6 *and *GJAI *genes (Fig .5C).

## Discussion

We compared different scaffolds by varying the electrospinning parameters to investigate the optimum parameters for a suitable cardiac scaffold. According to the SEM results of six scaffolds obtained from different electrospinning parameters listed in Table 1, the following observations were made: i) a decrease in the polymer feeding rate along with an increase in the distance between the needle and collector resulted in dramatic reduction in the nanofibre diameters in addition to a partial loss in homogenization of the fibres (Samples A1 and A2) (30). ii) When the voltage was decreased, the nanofibres with high discrepancy and a non-homogenized distribution were produced. This observation indicated that the imposed voltage was not suitable to generate a Taylor cone in the mentioned electrospinning process (Samples A1 and A2) (30). iii) When SEM images were studied based on Mandrel rotation speed, it was concluded that increasing the Mandrel rotation speed to a value over its threshold did not result in more aligned nanofibres. The high speed of the mandrel caused the polymer to spread around the collector, which resulted in non-homogenous distribution of the nanofibres (sample B3). iv) Conversely, when the Mandrel rotation speed was less than the threshold, we obtained a scaffold with a weak alignment (sample B1). Therefore, sample B2 was selected as the optimum sample based on the SEM images from all of the samples.

In order to achieve the best composite proportion, the prepared scaffolds were studied based on their mechanical strength and cellular adhesion properties. As expected, evaluation of the contact angle indicated that increasing the rate of the hydrophilic polymer (gelatine) resulted in a decreased contact angle and increased cellular adhesion (18). As indicated in Stress-Strain graph, when the PCL rate increased, the slope of the stress-strain plots and elasticity modulus were also elevated (18, 37). The fracture point in the scaffold composited with a higher PCL ratio occurred when a higher tension percentage was applied (37). Therefore, the results appear to be promising for future advances with the mechanical loading imposed with a 10% strain on the scaffold. Due to the results of MTS assay, the number of cells on the scaffolds increased over time. It was found that integration of gelatine led to an increase in cellular adhesion on the PCL/gelatine composite scaffold compared to the control scaffold. 

Eventually, according to all the tests performed on the scaffold, it was clear that we achieved a proper cardiac scaffold; therefore, the scaffold could be subjected to mechanical loading. The dynamic scaffold after five days of simulation was compared with the static scaffold. As shown in SEM micrographs of CPCs, the number of cells grown on the scaffold with the dynamic culture conditions was increased. Quantitative real-time PCR analysis indicated that the cardiac genes were expressed more in the dynamic scaffold compared with the static scaffold. *TTN* and *MYH-6* are transcribed to the Titin protein and α-MHC, respectively, which are responsible for cardiac muscle contraction. *GJAI *is transcribed to Connexin-43, which is a Gap junction protein responsible for regulating intercellular relations and synchronized cardiac contraction (25, 35). Our results elucidated that the expressions of the *TTN, MYH-6* and *GJAI* genes increased in the scaffold with the mechanical loading profile compared to the static culture condition. This finding indicated the appropriate transfer of tension force to the cardiomyocytes in the scaffold that had a mechanical loading profile. The dynamic condition induced higher gene expressions that were related to the transfer of a contractile force through natural cardiac tissue. 

## Conclusion

The goal of our study was to appropriately simulate and mimic cardiac ECM and the mechanical conditions in the heart tissue *in vitro*. We used an electrospun scaffold with aligned nanofibres combined with two PCL and gelatine polymers and produced a scaffold with suitable cellular adhesion and mechanical strength. The resultant scaffold showed a homogenous and consistent diameter distribution with a chemical-physical profile similar to cardiac ECM. Next, an MLD was used to produce a 10% strain with 1 Hz frequency to CPCs seeded on the scaffold for five days in the direction of the parallel nanofibres. This established a similar condition to the heart muscle with simultaneous contraction among cells with mechanical loading transferred through Gap junctions. Based on physics theories, applying a mechanical force in a special direct way would allow it to transmit more efficiently, such that the applied stress to the 2D aligned nanofibre scaffold would stimulate the CPCs to express more cardiac genes. Therefore, the relevant genes that are responsible for synchronized cardiac contraction and regular intercellular relationship (*MYH-6, TTN* and *CX43*) could be expressed at higher levels in these cells. Finally, these cells would be suitable candidates for transplantation to the damaged heart tissue without the possibility of developing arrhythmias. A relevant future topic could focus on the effect of infrared radiation from a non-contact sensor applied in the thermal control system in this project. According to a study by a research team at Utah University in 2011, infrared radiation was used to stimulate neonatal rat ventricular cardiomyocytes and toadfish middle ear cells to send neural signals to the brain.
